# Indoor air quality in day-care centers, kindergartens, and primary schools: a pediatric health-oriented operational framework aligned with European and U.S. IAQ standards

**DOI:** 10.3389/fped.2026.1801649

**Published:** 2026-06-16

**Authors:** Carmen Díaz-López, Alejandro Morales-Ruiz, María Dolores Joyanes-Díaz, Carmen M. Muñoz-González

**Affiliations:** Departamento de Arte y Arquitectura, Universidad de Málaga, Málaga, Spain

**Keywords:** ASHRAE, children, CO_2_, dampness, day-care, indoor air quality, kindergarten, Level(s)

## Abstract

**Background:**

Children aged 0–12 spend many hours in day-care and school buildings characterized by high occupant density, synchronized occupancy peaks, intense activity, and frequent transitions. Exposure patterns differ between early childhood settings (day care/kindergarten/preschool) and primary schools due to differences in schedules, floor-level activity, cleaning routines, and staff-mediated operations. However, recent evidence remains heterogeneous and is often interpreted without sufficient differentiation between educational levels or adequate emphasis on operational verification under real-world conditions.

**Objectives:**

This study aims to (O1) synthesize recent *in situ* evidence on IAQ in day-care, kindergarten/preschool, and primary school settings; (O2) identify determinants explaining exposure patterns (D1, occupancy/use intensity; D2, ventilation strategy and real operation; D3, seasonality and comfort-driven rebound; D4, outdoor pollution context, D5 envelope–moisture pathway); and (O3) translate the evidence into a conceptual, verifiable N1–N3 pathway aligned with European and U.S. normative anchors.

**Methods:**

PRISMA 2020 reporting was followed. Searches in Scopus, Web of Science, and PubMed (2018–2025; last search conducted on 12 September 2025) identified 990 records (Scopus, 345; Web of Science, 456; PubMed, 189). After removing 289 duplicates, 701 records were screened, and 614 were excluded based on title and abstract. Reports assessed for eligibility (*n* = 87) led to the exclusion of 61 studies with documented reasons, leaving 26 studies included in the qualitative synthesis. Data were managed in Excel with audit fields and standardized operational metrics (in-occupancy P90, percentage of time out of range, exceedance duration, pollutant peaks, and humidity risk-time indicators). Educational level, ventilation regime, season, and urban/outdoor context were explicitly coded. For ease of traceability, study characteristics are reported in the main text.

**Results:**

Recurrent CO_2_ exceedances were common when ventilation depended on occupant behavior, particularly in winter, when thermal comfort constraints limited window opening. Across the included studies and recent school-based correlation literature, CO_2_ emerged as a robust indicator of occupancy-linked ventilation performance; however, it is not as a standalone surrogate for outdoor-dominated pollutants such as NO_2_ or for all PM/VOC behaviors. Early childhood settings showed a stronger dependence on staff-mediated routines, cleaning schedules, and floor-level activity, whereas primary classrooms exhibited clearer accumulation during teaching blocks and greater room-to-room variability driven by timetables and teacher practices. Evidence on moisture, condensation, and mold was more limited and methodologically less standardized than the CO_2_/ventilation evidence base; therefore, related recommendations are framed as precautionary and escalation-oriented rather than as equally validated across all contexts.

**Conclusion:**

The manuscript's specific contribution is to reinterpret 26 recent *in situ* studies through an IAQ-centered, determinant-based lens that explicitly differentiates educational levels and translates heterogeneous findings into a conceptual, verifiable N1–N3 pathway. The review indicates that pediatric-relevant IAQ management should combine ventilation, filtration, source control, and moisture-risk mitigation and that performance should be verified using in-occupancy, episode-sensitive metrics rather than daily averages alone. The N1–N3 structure is presented as evidence-based implementation guidance derived from the review corpus and normative cross-checking; however, it requires further validation in applied school settings.

## 1 Introduction

The review is framed as an IAQ-centered synthesis within the broader IEQ field. It distinguishes early childhood settings from primary schools and explains why school-specific exposure dynamics require operational metrics and interventions tailored to children's schedules and vulnerabilities.

Indoor environmental quality (IEQ) in educational settings is increasingly recognized as a modifiable determinant of pediatric health and well-being. However, IEQ formally includes indoor air quality (IAQ), thermal comfort, lighting, and acoustics under EN 16798-1, whereas the contemporary monitoring literature synthesized in this paper is primarily focused on air quality and only secondarily reports temperature and relative humidity as contextual variables (25, 53). For this reason, the revised manuscript explicitly adopts IAQ as its main focus, while retaining hygrothermal and moisture-related variables where they shape ventilation operation or dampness risk. Children spend many hours each weekday in day-care and school environments, resulting in exposures that are repeated, cumulative, and operationally tractable through building operations and targeted upgrades.

It is also necessary to distinguish early childhood settings from primary schools. Day-care centers and kindergartens/preschools involve younger children, floor-level contact, nap or rest periods, greater staff mediation, more intensive cleaning and product use, and often more conservative thermal management by caregivers. Primary school classrooms, by contrast, typically involve longer seated teaching blocks, higher room occupancy, clearer schedule-driven CO₂ accumulation, and teacher-managed window-opening practices. These differences do not justify splitting the review into distinct manuscripts, as many building and ventilation determinants are shared; however, they do require differentiated interpretation of routines, pollutant peaks, and feasible interventions (1, 2, 15, 28, 30–33, 36, 37, 40–49, 51).

IAQ also influences learning-related outcomes. A consistent body of literature links inadequate classroom ventilation and poor environmental conditions to with evidence that ventilation and thermal conditions act jointly rather than independently (3–9, 14). This interaction is decisive for implementation: interventions based solely on manual window opening can fail when opening windows leads to cold discomfort, drafts, rain exposure, noise intrusion, or overheating. Importantly, any resulting rebound pattern is context-dependent rather than universal or purely behavioral. It depends on age group, staff practices, occupancy density, climatic stress, and outdoor conditions. Recent school studies indicate that naturally ventilated rooms often alternate between acceptable conditions during mild periods and repeated deterioration during winter or pollution episodes, especially when ventilation remains strongly dependent on staff or teacher behavior (29, 36, 39, 42, 43, 46, 47, 51).

Since 2020, low-cost CO₂ monitoring has expanded in educational buildings. CO₂ is a useful operational proxy because its dominant classroom source is metabolic, and its concentration reflects the balance between occupant emissions and dilution by ventilation. It can be related to rebreathed air fraction and has been used to estimate airborne infection risk and guide ventilation management (10–13). However, recent classroom reviews and multipollutant studies reinforce a crucial limitation: while CO₂ is informative for occupancy-linked ventilation performance, correlations with other indoor pollutants are conditional rather than universal. CO₂ tends to covary more consistently with occupancy-generated or ventilation-mediated patterns than with traffic-related NO₂, episodic VOC emissions, or all PM fractions, which are strongly shaped by outdoor burden, resuspension, source events, and timing (16, 18, 45, 47, 49, 52). Therefore, CO₂ should be treated as a necessary but insufficient anchor metric within a broader IAQ strategy.

School IAQ evidence increasingly supports an outdoor-context approach. Multicountry school monitoring has documented the coexistence of ventilation-related CO₂ challenges with particle and comfort concerns, reinforcing the need for integrated, multidomain strategies rather than single-parameter management (15). In addition, dedicated school monitoring initiatives have operationalized low-cost protocols to enable scale-up and equity-sensitive prioritization at the portfolio level (17). Catalonia's Sentinel Schools Network monitored CO₂ and NO₂ across multiple periods, showing that ventilation practices and traffic-related burdens jointly shape indoor exposure profiles (18, 19). However, explicit urban–rural comparisons remain scarce, and much of the recent evidence comes from urban or metropolitan schools; therefore, context must be interpreted carefully rather than generalized mechanically across all school types.

To translate IAQ evidence into measurable and auditable actions, governance and verification frameworks are needed. European references offer complementary functions: Level(s) Indicator 4.1 structures IAQ strategy and use-phase verification; EN 16798-1 and its interpretation guide CEN/TR 16798-2 define indoor environment categories and input parameters; EN 16798-3 provides system-level performance requirements for non-residential ventilation; and Spain's RITE provides a regulatory baseline for operation, maintenance and thermal system performance (23–26, 53, 54). In the United States, ANSI/ASHRAE Standard 62.1 provides minimum ventilation and acceptable IAQ requirements for non-residential buildings, while WHO pollutant guidelines offer health-based benchmarks for PM, NO₂, and selected indoor pollutants that are useful when ventilation metrics alone are insufficient (12, 21, 55, 56). These documents are similar in recognizing ventilation, source control, and operation/maintenance as core levers but differ in emphasis: European documents place greater emphasis on category-setting and verification architecture, ASHRAE 62.1 focuses more on ventilation system requirements, and WHO guidance prioritizes pollutant-specific health protection.

Accordingly, the revised manuscript pursues three linked objectives: (O1) synthesize recent *in situ* IAQ evidence in day-care, kindergarten/preschool, and primary school settings for children aged 0–12; (O2) identify operational and contextual determinants explaining exposure patterns; and (O3) translate the evidence into a conceptual, verifiable N1–N3 intervention pathway aligned with European and U.S. normative anchors. The manuscript's original contribution lies not in restating that school air quality matters but in integrating heterogeneous monitoring studies into a determinant-based, pediatric-relevant, and implementation-oriented framework that explicitly differentiates educational levels and prioritizes episode-sensitive metrics over daily averages. The proposed N1–N3 pathway is intended primarily as conceptual and operational guidance and should not be interpreted as a fully validated decision framework.

## 2 Theoretical framework and normative alignment

This section introduces the determinant model (D1–D5) used to interpret study findings, justifies the selection of operational metrics for school IAQ management, and outlines the normative anchors used to translate evidence into portfolio-ready actions.

### 2.1 Determinant model (D1–D5)

We conceptualize school IAQ as the interaction of sources, transport and mixing, ventilation and infiltration, and removal or transformation, shaped by behavioral routines, outdoor burden, and building condition. The five determinants were selected because they represent the causal structure most repeatedly observed across the 26 included studies and because each determinant corresponds to a decision-relevant lever for diagnosis or intervention. D1 (occupancy and use intensity) captures density, schedules, activity, and resuspension potential; D2 (ventilation strategy and real operation) captures natural, mechanical, or hybrid systems, window regimes, commissioning, and maintenance; D3 (seasonality and comfort-driven rebound) captures the recurrent deterioration associated with climatic stress and acceptability constraints; D4 (outdoor pollution context) captures traffic, urban burden, and episodic outdoor air quality; and D5 (envelope–moisture pathway) captures thermal bridges, cold surfaces, water ingress, persistent humidity, and mold risk. Together, these determinants are not intended as an exhaustive model of all IEQ domains but as an IAQ-centered interpretive scaffold with direct implementation relevance.

### 2.2 Operational metrics

Operational metrics were selected on three grounds: pediatric relevance, compatibility with the school day, and transferability to building management and postoccupancy verification. Educational exposures are episodic and schedule-driven; therefore, daily averages can obscure the peaks that matter most during occupied teaching blocks. For CO₂, in-occupancy P90, percentage of time above trigger levels during occupancy, exceedance duration, and maximum exceedance capture both intensity and persistence. For particles and VOCs (or TVOC), peak frequency and peak duration during occupancy are emphasized, with explicit attention to events such as arrivals, play, cleaning, and purge ventilation. For humidity, percentage of time within moisture-risk RH bands and co-occurrence with low-ventilation signatures are used to represent combined ventilation–moisture risk. These choices are consistent with an IAQ-centered reading of Level(s), EN 16798-1 and EN 16798-3, ASHRAE 62.1, and WHO pollutant guidance, all of which support decisions based on conditions actually experienced by occupants rather than off-hour averages (12, 23, 25, 54–56).

### 2.3 Normative anchors for translation

The proposed framework is aligned with Level(s) Indicator 4.1, which structures IAQ strategy and verification; EN 16798-1/UNE-EN 16798-1 and CEN/TR 16798-2, which define and interpret indoor-environment categories and input parameters; EN 16798-3:2025, which addresses ventilation system performance in non-residential buildings; Spain's RITE, which specifies minimum requirements for operation, maintenance, and performance of thermal installations; ANSI/ASHRAE Standard 62.1, which provides minimum ventilation and acceptable IAQ requirements; and WHO health-based air-quality guidance for pollutant interpretation where ventilation metrics alone are insufficient (21, 23–26, 53–56). Table 1 summarizes the main similarities and differences among these anchors as used in this review.

**Table 1:** Comparison of the normative anchors used to interpret school IAQ and translate findings into action.

Anchor	Type	Main use in this review	Similarity with other anchors	Key difference
Level(s) Indicator 4.1 (23)	EU use-phase framework	Structures IAQ strategy, logging, and verification	Recognizes monitoring, source control, and documentation	Framework-oriented rather than a stand-alone ventilation standard
EN 16798-1 + CEN/TR 16798-2 (25, 53)	Indoor environment categories and interpretation	Defines indoor-environment input parameters and category logic	Connects IAQ with thermal conditions and the broader indoor environment	Stronger on category setting than on detailed system requirements
EN 16798-3:2025 (54)	European non-residential ventilation system standard	Supports the interpretation of system performance and controlled solutions	Shares ventilation performance intent with ASHRAE 62.1	Focuses on system performance in non-residential buildings
RITE (26)	Spanish regulatory baseline	Frames operation, maintenance, and compliance in Spain	Emphasizes operation and maintenance alongside ventilation	National regulatory scope rather than an international comparison standard
ANSI/ASHRAE 62.1 (55)	U.S. ventilation and acceptable IAQ standards	Provides minimum ventilation and acceptable IAQ requirements	Shares focus on ventilation, filtration, and operations	More prescriptive on ventilation system requirements than Level(s)
WHO air-quality guidance (21, 56)	Health-based pollutant guidance	Supports the interpretation of PM, NO₂, and pollutant risk when CO₂ is insufficient	Complements source control and ventilation logic	Health-benchmark orientation rather than building-design procedure

## 3 Materials and methods

This section describes the systematic review workflow (search, screening, and extraction) and the analytic choices that connect the review to implementation. Reporting follows PRISMA 2020 guidelines (27).

### 3.1 Eligibility criteria

The review question was structured using the PICOS framework to align with systematic review reporting requirements: population = children aged 0–12 in day-care, kindergarten/preschool, and primary school settings; intervention/exposure = real-world IAQ conditions and building operation determinants, including ventilation regimes, source-control practices, and contextual stressors; comparator = contrasts across educational levels, ventilation modes, seasons, and outdoor contexts, where reported; outcomes = IAQ pollutants/proxies and operational metrics relevant to pediatric exposure (e.g., CO_2_, PM, NO_2_, VOCs/TVOC, and humidity/moisture indicators); and study design = *in situ* monitoring studies conducted in real educational settings. This PICOS framing guided eligibility, extraction, and the structured narrative synthesis.

Inclusion criteria were (i) *in situ* monitoring in real day-care, kindergarten/preschool, or primary school settings serving children aged 0–12; (ii) reporting of at least one IAQ pollutant or ventilation proxy (CO₂, PM₂.₅ or PM₁₀, NO₂, VOCs or TVOC, formaldehyde, bioaerosol or fungal contamination, or indicators of dampness, condensation, or mold), with temperature and relative humidity accepted as contextual variables when paired with IAQ interpretation; (iii) minimum methodological transparency (monitoring period, sampling interval or campaign duration, and basic instrument description); and (iv) minimum operational context (educational level, occupancy schedule, and/or ventilation regime). Because the revised review is IAQ-centered, studies focused solely on lighting, acoustics, or thermal comfort without extractable IAQ information were considered outside the scope rather than treated as negative evidence.

### 3.2 Information sources and search strategy

Searches were conducted in Scopus, Web of Science Core Collection, and PubMed between 2018 and 2025 (last search: 12 September 2025). Search strings combined four concept blocks: (i) educational setting (day care, childcare, nursery, kindergarten, preschool, primary school, classroom); (ii) IAQ domains (indoor air quality, CO₂, ventilation, particulate matter, PM₂.₅, PM₁₀, NO₂, VOC, TVOC, formaldehyde, bioaerosols, dampness, condensation, mold); (iii) child population; and (iv) educational buildings. The full database-adapted strings are provided in Supplementary Table S2.

### 3.3 Record management, deduplication, and screening

Records were exported and consolidated in an Excel master workbook with an audit trail. Deduplication was performed using DOI or PMID matching, where available, along with normalized title checks. Duplicates were flagged rather than deleted to preserve traceability. Screening was performed in two stages (title or abstract, followed by full text) against the eligibility criteria, and each full-text exclusion was assigned a prespecified operational reason in the audit workbook.

### 3.4 PRISMA flow and final counts

A total of 990 records were identified (Scopus, 345; Web of Science, 456; PubMed, 189). A total of 289 records were removed before screening as duplicates. A total of 701 records were screened. A total of 614 records were excluded at the title/abstract stage. A total of 87 reports were assessed for eligibility (all retrieved). A total of 61 reports were excluded with documented reasons: *n* = 61. Finally, 26 studies were included in qualitative synthesis. The documented reasons for full-text exclusion are summarized in Table 1.

**Figure 1:**
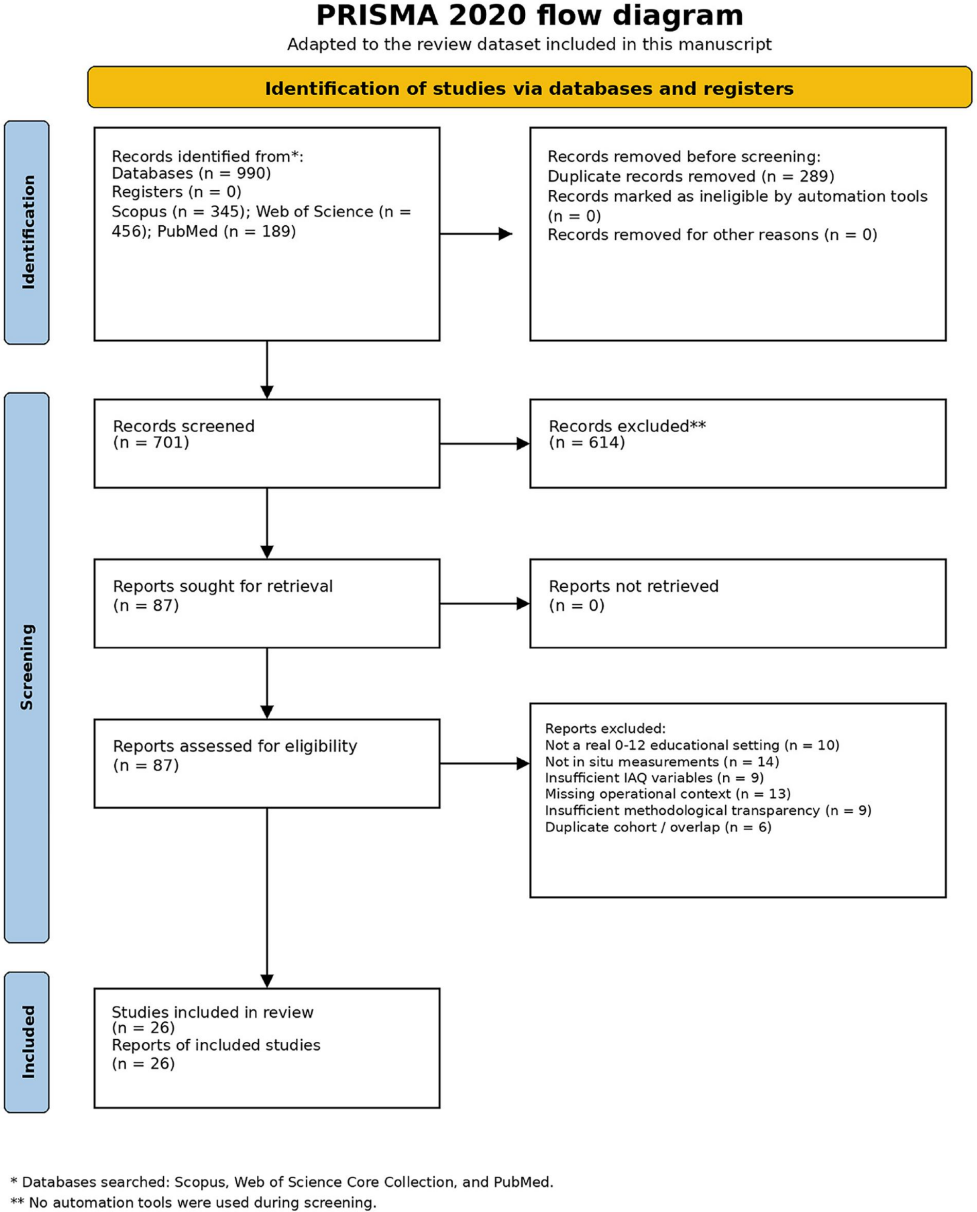
PRISMA 2020 flow diagram (final counts).

**Figure 2:**
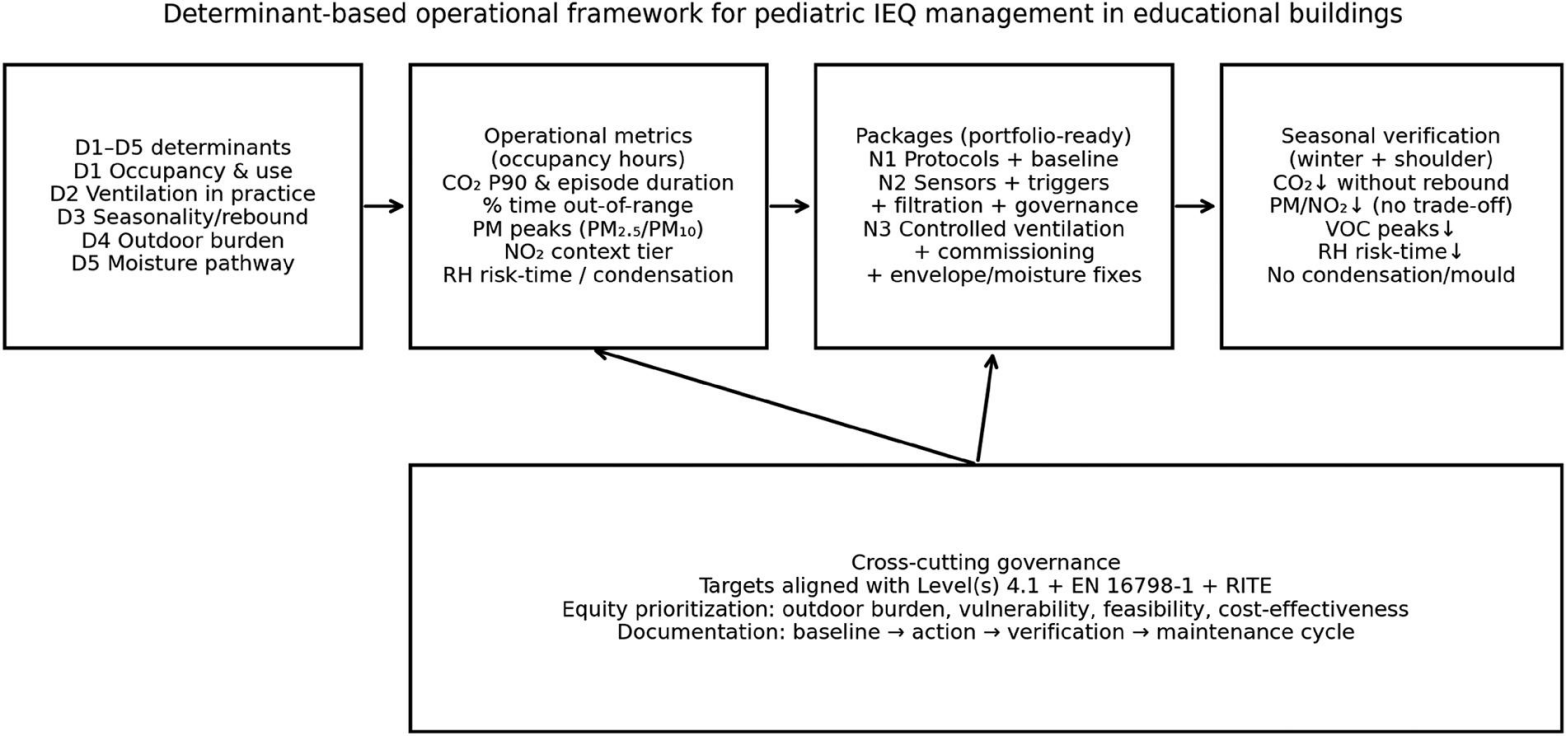
Authors' conceptual synthesis of determinant coding and thematic grouping across the 26 included studies, showing how D1–D5 inform N1–N3 packages and seasonal verification.

Figure 1 presents the record identification and screening workflow. The conceptual relationships between determinants, metrics, and intervention packages are presented later, after the synthesis of results, in Figure 2. A summary of the proposed N1–N3 package pathway, determinant targets, and seasonal verification endpoints is presented in Table 2.

**Table 2:** N1–N3 package pathway with seasonal verification endpoints and representative supporting studies.

Tier	Core actions	Primary determinant targets	Minimum verification set (seasonal)	Representative supporting studies
N1	Baseline (CO₂ + T/RH) + routines + source control	D1; D2 (partial); D5 (screen)	CO₂ P90↓; episode duration↓; RH risk-time↓	18, 31, 37, 39, 41, 44, 49, 51
N2	Visible sensors + triggers + filtration + accountability	D1–D4; D5 (early control)	CO₂ P90↓; PM peaks↓; variability↓; RH risk-time↓	18, 34, 35, 38, 39, 42, 48, 50
N3	Controlled ventilation + commissioning + envelope/moisture remediation	D2–D5	Winter robustness; PM↓ without CO₂ rebound; no condensation	20–22, 29, 36, 43, 46, 47

### 3.5 Data extraction, determinant coding, and synthesis

A standardized extraction sheet captured study citation, country or region, setting type, educational level, season, sample size, building and system characteristics, ventilation strategy, operational profile (occupancy and routines), monitoring design (instrumentation, interval, duration), IAQ domains, and main findings. Data abstraction also recorded urban or metropolitan context when explicitly reported, as well as whether the study allowed indoor–outdoor interpretation. Characteristics of the 26 included studies are summarized in Supplementary Table S1 and reproduced in Supplementary Appendix Table A1 for in-manuscript traceability. Determinants D1–D5 were coded for each study to support causal interpretation. Because monitoring protocols, pollutants, and reported metrics were heterogeneous, a structured narrative synthesis was used rather than meta-analysis. In this review, “documented reasons” for exclusion means that each full-text report was assigned a prespecified operational reason in the audit workbook rather than being discarded without a traceable label. “Insufficient IAQ variables” refers to studies that did not report an interpretable pollutant or ventilation-proxy dataset for the review question; “not *in situ* measurements” means simulation, chamber, or laboratory work without classroom validation; and “insufficient methodological transparency” refers to inadequate reporting of sensor description, sampling interval or duration, QA/QC, occupancy context, or ventilation context to support interpretation. To derive the N1–N3 pathway, we undertook an iterative thematic synthesis of intervention-relevant findings across the included studies: first, we mapped recurrent problems and signatures to determinants (for example, behavior-dependent CO₂ rebound, outdoor-driven PM or NO₂, cleaning-related VOC peaks, and moisture-risk indicators); second, we grouped reported or clearly implied responses by implementation depth; and third, we translated these groups into three escalating packages. N1 captures low-cost operational controls repeatedly supported across studies (baseline monitoring, routines, and source control). N2 captures measures that reduce behavioral dependence or mitigate outdoor burden without requiring full building retrofit (visible sensors, triggers, portable or local filtration, and governance). N3 captures structural or system-level measures indicated when recurrent failure persists across seasons or when condensation or mold risk points to building-level limits (controlled or hybrid ventilation, commissioning, envelope, and moisture remediation). Thus, the N1–N3 packages were not a pre-existing framework imported *a priori* but rather an analytic output of determinant coding, pattern comparison, and implementation-oriented synthesis across the review corpus.

### 3.6 Educational-level and context comparison strategy

Educational level was coded as early childhood education (day care and kindergarten or preschool), primary school, or mixed-school settings. Urban or metropolitan vs. other contexts, season, ventilation mode, and monitoring duration were also extracted when reported. Because subgroup sizes were unequal and many studies lacked one or more contextual descriptors, comparisons across educational levels and contexts were conducted as structured qualitative contrasts rather than formal subgroup effect estimates. In the Results and Discussion sections, differences are reported only where supported by the study findings or by repeated patterns across several studies.

## 4 Results

This section reports findings in a way that links the review corpus to study settings, determinants, and implementable actions. For readability, the determinant labels are repeated here: D1, occupancy and use intensity; D2, ventilation strategy and real operation; D3, seasonality and comfort-driven rebound; D4, outdoor pollution context; and D5, envelope–moisture pathway. The traceability between objectives, methods, and outputs is summarized in Table 3.

**Table 3:** Documented reasons for full-text (report) exclusion.

Reason for exclusion (reports)	Operational definition	*n*
Not a real 0–12 educational setting	Not day care/kindergarten/primary or not representative use	10
Not *in situ* measurements	Simulation/lab-only without classroom validation	14
Insufficient IAQ variables	No interpretable pollutant or ventilation-proxy dataset for an IAQ-centered synthesis	9
Missing operational context	No educational level, occupancy schedule, and/or ventilation regime information	13
Insufficient methodological transparency	Sensor/specification, sampling interval/duration, QA/QC, or operational context poorly described	9
Duplicate cohort/overlap	Same dataset without additional extractable outcomes	6
Total		61

**Table 4:** Traceability map linking objectives to methods and outputs.

Objective	Methodological phase	Key fields extracted/coded	Outputs in this paper
O1: Evidence synthesis	Selection + extraction	IAQ domains, setting type, educational level, season, and region	Sections 4.1–4.7; Supplementary Appendix Table A1
O2: Determinant identification	Determinant coding (D1–D5)	Operation, seasonality, outdoor context, envelope–moisture pathway	Sections 4.3–4.9
O3: Translation to action	Framework mapping	Metrics → packages; normative alignment	Section 4.9; Figure 2; Table 8

For ease of review, the study-level characteristics of all 26 included studies are reported in Supplementary Table S1 and reproduced at the end of the manuscript as Supplementary Appendix Table A1.

### 4.1 Study characteristics, educational levels, and contextual differentiation

The 26 included studies covered day-care centers, kindergartens or preschools, primary schools, and a small number of mixed-school settings; however, the distribution was uneven. Primary school environments dominated the evidence base, while day-care and preschool studies were fewer and were more often based on targeted campaigns or single-facility monitoring. Most studies were conducted in urban or metropolitan contexts and in naturally ventilated or behavior-dependent classrooms, whereas explicitly rural comparisons and fully mechanical school portfolios were relatively scarce (15, 18, 28–51).

These distributional features are important for interpretation. Early childhood settings are more strongly influenced by floor-level activity, staff-mediated operation, and cleaning or product schedules, while primary classrooms more often exhibit long accumulation during teaching blocks and greater room-to-room variability linked to teacher practices and class timetables. Because the underlying evidence is heterogeneous and subgroup sizes are unequal, these differences are interpreted qualitatively rather than as formal comparative effect estimates (31, 32, 37, 40, 41, 44, 45, 51).

### 4.2 Evidence landscape by IAQ domain

Across the included studies, CO₂ and thermal–hygrometric contextual variables were the most consistently reported, reflecting both their accessibility for monitoring and their centrality to ventilation and comfort assessment (15, 18, 28–51). Evidence on particle and NO₂ was more context-dependent and often relied on mixed monitoring approaches, including continuous classroom sensors, passive samplers, and targeted campaigns. Findings on VOCs were heterogeneous and strongly influenced by source events, product use, and timing. Evidence on moisture, condensation, and mold was the least standardized and should therefore be interpreted more cautiously than the better-supported CO₂ and ventilation findings.

### 4.3 CO₂: episodic dynamics, correlation limits, and decision metrics

**Figure 3:**
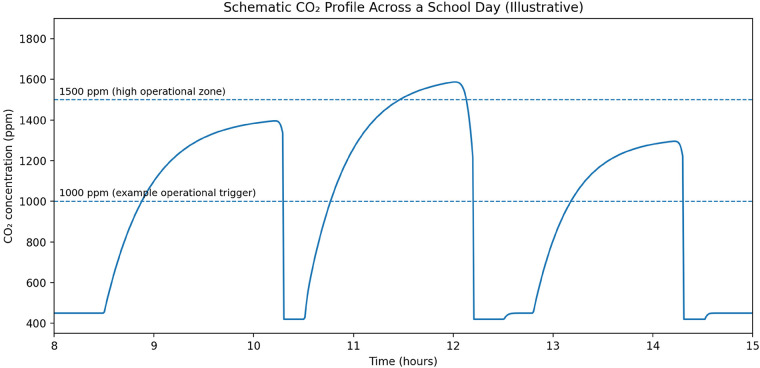
Schematic CO₂ profile across a school day, highlighting episodic exceedances and the importance of in-occupancy metrics.

CO₂ profiles in classrooms typically show rapid increases during teaching blocks and reductions during breaks or purge ventilation, with repeated occupancy-linked peaks documented across naturally ventilated and mixed-mode school settings (18, 28, 29, 33, 36, 39, 42–47, 51). Behavior-dependent natural ventilation is associated with greater room-to-room variability and longer exceedance duration during occupancy (29, 33, 36, 39, 45, 51). Seasonality is decisive: during winter, comfort constraints reduce the frequency and duration of window opening, leading to recurrent exceedances during the most critical periods (29, 36, 43, 46, 47, 51). These patterns support the use of in-occupancy P90 and exceedance duration rather than daily averages, as averages can conceal repeated high-exposure episodes (18, 28, 29, 42–47). At the same time, recent school correlation studies and multipollutant analyses indicate that CO₂ should not be interpreted as a universal surrogate for PM, NO₂, or VOCs: it most reliably diagnoses occupancy-linked dilution failure, while outdoor-driven or source-event pollutants require dedicated measurement or contextual indicators (16, 18, 45, 47, 49, 52). Figure 3 illustrates this episode-sensitive interpretation, and Table 4 summarizes the corresponding CO_2_ decision logic.

### 4.4 Ventilation–comfort tradeoff and rebound

Ventilation performance in educational settings is constrained by comfort and acceptability. When ventilation requires window opening, decisions are influenced by cold discomfort, drafts, rain, noise intrusion, and safety considerations, and several school studies report operational deterioration when these constraints accumulate during occupied hours (29, 33, 36, 39, 42, 43, 46, 47, 51). A rebound pattern is observed when windows are closed during high-occupancy periods and opened later to recover comfort. This rebound should not be interpreted as a simple behavioral failure; rather, it reflects the interaction among user practices, building-system limitations, seasonal stress, and outdoor conditions. In early childhood settings, caregivers may prioritize draught avoidance, nap comfort, and thermal stability for children, whereas in primary classrooms, teacher practices and longer teaching blocks tend to dominate. Persistent seasonal rebound therefore supports escalation to controlled ventilation and commissioning (N3), especially when the goal is to maintain ventilation during winter without compromising comfort (29, 31, 36, 39, 42, 43, 46, 47, 51).

### 4.5 Particulate matter: outdoor burden, ingress, and resuspension

Particle exposure patterns reflect both outdoor ingress (D4) and indoor resuspension (D1). PM₂.₅ is frequently influenced by ambient outdoor levels in naturally ventilated schools or urban child-care settings, whereas PM₁₀ often exhibits activity-linked peaks due to resuspension during movement, play, and cleaning (15, 28, 30, 32–39, 41, 42, 45, 46, 48, 50). Several studies also highlight a practical CO₂–PM tradeoff: increasing outdoor air exchange can reduce CO₂ levels while increasing particulate ingress during outdoor pollution episodes or in traffic-affected locations (32, 35, 38, 39, 42, 45, 48). Where urban, roadside, or alert-day contexts were explicitly described, this tradeoff became more acute, supporting the use of filtration and time-adaptive ventilation strategies rather than “more ventilation” as a universal strategy (34, 35, 38, 39, 42, 48, 50). Table 5 summarizes the particle-management logic.

**Table 5:** CO₂ decision logic: determinants, signatures, metrics, actions, and representative supporting studies.

Determinant pathway	Operational signature	Why daily averages fail	Recommended metric(s)	Most direct action	Representative supporting studies
D1 + D2	Rapid accumulation during occupancy	Off-hour dilution reduces the mean	CO₂ P90 (occupancy); episode duration	Optimize routines; reduce behavioral dependence	18, 28, 29, 33, 36, 39, 42–47, 51
D3	Winter deterioration vs. mild seasons	Annual mean masks seasonal failure	Percentage of time out of range by season	Comfort-compatible ventilation; consider controlled solutions	29, 36, 43, 46, 47, 51
D2	Room-to-room variability	Building mean hides hotspots	Room-level % out of range; variability index	Governance + feedback; target critical rooms	29, 33, 39, 42, 45, 51

### 4.6 NO₂: context tiering and prioritization

NO₂ is a marker of traffic-related outdoor burden and supports context tiering across school portfolios (18, 30, 35, 37, 41, 45, 48). Catalonia's Sentinel Schools Network documented NO₂ alongside CO₂ across multiple time periods, showing that ventilation practices and outdoor burden jointly shape indoor exposure profiles (18). Comparable findings from day-care, nationwide school, and urban classroom studies indicate that NO₂ often reflects outdoor context more strongly than indoor generation, making location-sensitive prioritization essential (30, 37, 41, 45, 48). This also reinforces a methodological point: achieving a nominal CO₂ target does not guarantee protection from outdoor-derived pollutants. In high-burden contexts, protective strategies should therefore combine filtration, careful ventilation timing, and, where relevant, intake and enclosure considerations. Context tiering also supports equity-oriented prioritization: schools with higher outdoor burdens require stronger packages to achieve comparable indoor protection (18, 35, 45, 48, 56).

### 4.7 VOCs and TVOC: episodic peaks and source control

VOCs (or TVOC) commonly exhibit episodic peaks associated with cleaning events, product choices, storage conditions, and off-gassing from materials, although baseline VOC mixtures also vary by building age, furnishings, and ventilation strategy (30, 31, 37, 40, 41, 43–49). In day-care and preschool settings, higher cleaning frequency, increased handling of toys and materials, and staff-managed routines can increase emission potential or shift the timing of peaks (31, 37, 40, 41). Source control is therefore a first-line intervention: using low-emission products and materials, safe storage practices, and scheduling cleaning activities outside occupancy with postcleaning purge ventilation (37, 41, 43, 44, 48, 49). Where peaks persist, visible feedback and standardized governance can reduce recurrence by aligning routines across classrooms and strengthening adherence to ventilation or filtration protocols (39, 42–44, 49, 51). Table 6 summarizes the VOC/TVOC decision logic.

**Table 6:** Particle management: drivers, metrics, packages, verification, and representative supporting studies.

Dominant driver	Typical signal	Decision metric(s)	Preferred package	Verification (avoid trade-off)	Representative supporting studies
Outdoor burden (D4)	PM tracks outdoor pollution; it rises during episodes	PM₂.₅ P90; peak episodes; context tier	N2 filtration + adaptive ventilation	PM↓ without CO₂ rebound	18, 30, 35, 38, 41, 45, 48, 50
Resuspension (D1)	PM₁₀ peaks during activity/cleaning	Peak count/duration by schedule	N1 protocols; N2 if persistent	Peak frequency↓	15, 28, 30, 32, 36, 39, 46
Infiltration/envelope (D2)	Elevated baseline even when closed	Baseline indicator	N3 envelope + controlled ventilation/filtration	Baseline↓	35, 38, 45, 48, 50

### 4.8 Moisture, condensation, and mold pathway

Moisture and mold constitute a pediatric-critical pathway, but they were also the least consistently characterized domains in the included studies. When reported, risk signals generally appeared as persistent high RH, dampness-related building characteristics, fungal or biological contamination, or visible deterioration rather than through harmonized mold metrics (15, 30, 41, 49). Within this evidence base, these indicators support treating prolonged RH persistence, recurrent condensation, cold corners, and visible mold as D5-driven warning signs that justify precautionary escalation, particularly when they recur across seasons or coexist with poor ventilation signatures. Operational measures (N1–N2) may reduce risk when RH elevation is moderate and reversible; however, recurrent condensation, dampness-associated building defects, or biological contamination indicate the need for N3-level causal remediation. Because the underlying evidence base is relatively limited and methodologically uneven, these recommendations should be read as pragmatic and preventive rather than as definitively validated thresholds. Table 7 summarizes the moisture pathway indicators and escalation logic.

**Table 7:** VOC or TVOC decision logic, verification, and representative supporting studies.

Scenario	Operational signature	Primary measure(s)	Package level	Verification metric	Representative supporting studies
Cleaning-related peaks	TVOC peaks after cleaning	Product substitution + schedule + purge	N1	Peak height/frequency↓	31, 37, 40, 41, 43, 44, 48, 49
Persistent elevation	Sustained higher baseline	Identify sources/storage; improve effective ventilation	N1 → N2	Baseline/persistence↓	30, 31, 40, 41, 45, 49
Peaks coincide with high CO₂	Accumulation under low ventilation	Triggers + protocols + feedback	N2	Peak duration↓	41, 45, 47, 49

### 4.9 Translation to N1–N3 packages and seasonal verification

The determinant-based synthesis supports a three-tier intervention pathway for portfolio deployment. N1 focuses on rapid, low-cost controls (protocols, baseline monitoring, and source control). N2 adds measures that reduce behavioral dependence and mitigate outdoor pollution exposure (visible sensors, triggers, portable or local filtration where needed, and governance). N3 addresses structural limitations (controlled ventilation or hybrid solutions with commissioning and, where relevant, heat recovery to reduce winter rebound; envelope and thermal bridge remediation to prevent condensation and mold). Seasonal verification (winter plus at least one shoulder season) is required to demonstrate that improvements persist under stress conditions. Figure 3 represents the authors' own conceptual synthesis derived from determinant coding and thematic synthesis of the 26 included studies; it is therefore presented as a postresults interpretive framework rather than a pre-existing or formally validated operational tool. The logic linking evidence, risk, action, and verification was built from repeated patterns observed across the review corpus and then cross-checked against the normative anchors summarized in Section 2.3 (18, 29, 35, 38, 39, 42, 43, 46, 48, 50, 51). Future application studies should evaluate its usability, reproducibility, and decision-making value in real-world school settings. Table 8 summarizes the N1–N3 package pathway and seasonal verification endpoints.

**Table 8:** Moisture pathway indicators, escalation logic, and representative supporting studies.

Evidence	Risk interpretation	Action logic	Package level	Verification endpoint	Representative supporting studies
High RH risk-time (no visible mold)	Moderate moisture risk	Strengthen ventilation + routines	N1 → N2	RH risk-time↓	15, 41, 46, 47
Recurrent condensation	High mold risk	Address surfaces + ventilation control	N3	Condensation eliminated	15, 30, 41, 49
Visible mold/odor	Very high risk	Urgent causal remediation + prevention plan	N3 (immediate)	No recurrence + prevention plan in place	15, 30, 41, 49

## 5 Discussion

The revised synthesis supports an IAQ-centered, rather than a generic IEQ, interpretation of the evidence. This is important because the included studies predominantly monitored CO₂, PM, NO₂, VOCs, and temperature or relative humidity, whereas lighting and acoustics were not part of the formal corpus. The contribution of the present review is therefore to make this scope explicit and to avoid over-claiming full IEQ coverage. At the same time, retaining temperature, RH, and moisture indicators remains necessary because they mediate ventilation acceptability and mold risk, both of which directly affect IAQ performance (25, 53, 54).

A second interpretive point concerns educational levels. The review lacked a sufficiently balanced corpus for formal subgroup-effect estimation, yet consistent qualitative differences emerged. Early childhood settings showed greater sensitivity to cleaning and product-use schedules, staff-mediated ventilation, and child comfort constraints, whereas primary classrooms more often showed teaching-block accumulation, room-to-room variability, and recurrent winter rebound. These differences justify differentiated governance and monitoring protocols, even when the same determinant model is applied across all schools (29, 31, 37, 40, 41, 43, 45, 51).

Third, the results clarify the strengths and limitations of CO₂ as an indicator. CO₂ is highly valuable for diagnosing ventilation relative to occupancy and for identifying critical rooms and time periods; however, it is not a global IAQ indicator. The review and recent school-based correlation literature consistently show that PM and NO₂ are often dominated by outdoor burden and resuspension, while VOCs reflect product use and material-related events. Consequently, a CO₂-centric strategy can under-protect children in roadside schools or in settings with recurrent cleaning-related peaks unless it is combined with pollutant-specific or context-sensitive controls (16, 18, 45, 47, 49, 52, 56).

Fourth, the comparison of standards has practical implications. European documents offer a strong architecture for category-setting and verification across the life cycle and use phase, whereas ASHRAE 62.1 provides a stronger foundation for minimum ventilation and system requirements. WHO guidance, by contrast, is most useful when interpreting pollutant concentrations from a health-protection perspective. Using these references in combination is more informative for schools than relying on a single CO₂ benchmark or a single ventilation-rate target (21, 23–26, 53–56).

Finally, the evidence base remains uneven. Most recent studies were conducted in urban or metropolitan settings, and many focused on naturally ventilated or behavior-dependent classrooms. Rural contexts, explicitly mechanical school stock, standardized mold metrics, and directly comparable day-care vs. primary-school datasets were less common. Future studies would be strengthened by standardized reporting of educational level, occupancy profile, season, ventilation mode, outdoor context, and episode-sensitive metrics, as well as by more robust and harmonized assessment of moisture and mold pathways.

## 6 Conclusions

This review makes three specific contributions. First, it correctly reframes the recent evidence base as IAQ-centered, while clarifying that temperature and relative humidity function mainly as contextual variables influencing ventilation operation and moisture risk rather than as indicators of full IEQ coverage. Second, it differentiates early childhood settings from primary schools within a common determinant model, showing that staff-mediated operation, cleaning routines, and floor-level activity are especially relevant in day-care or preschool settings, whereas long teaching-block accumulation and room-level variability are especially relevant in primary classrooms. Third, it translates heterogeneous recent monitoring evidence into a conceptual, verifiable N1–N3 pathway aligned with European and U.S. normative anchors.

From a practical standpoint, the evidence indicates that CO₂ should be used as a ventilation performance indicator, rather than as a stand-alone surrogate for all pollutants; that PM and NO₂ management must account for outdoor burden and infiltration; that VOC control requires source and schedule management; and that recurrent condensation or dampness requires causal, building-level remediation. Figure 3 should therefore be interpreted as the authors' evidence-based synthesis for implementation rather than as a fully validated operational framework. Future school IAQ studies and policies should report and verify performance by occupied period, educational level, season, and context, rather than relying on single annual averages or generic school-wide assumptions.

## Data Availability

The original contributions presented in the study are included in the article/Supplementary Material, further inquiries can be directed to the corresponding author.
