# Supplementary material for: Indoor air quality in day-care centers, kindergartens, and primary schools: a pediatric health-oriented operational framework aligned with European and U.S. IAQ standards
Source: Front Pediatr. 2026 Jun 16;14:1801649. doi: 10.3389/fped.2026.1801649 (PMC13325463; doi:10.3389/fped.2026.1801649)
Supplement: Supplementary file 1 [file Table1.docx]

**Appendix A. In-manuscript study-characteristics table**

Table A1. Characteristics of the 26 included studies.

| **Ref** | **Included study** | **Country** | **Setting** | **Sample size** | **IEQ parameters measured** | **Ventilation type** | **Monitoring duration** |
| --- | --- | --- | --- | --- | --- | --- | --- |
| 15 | Baloch et al., 2020 | 23 European countries | Primary schools | 115 schools; 319 classrooms; 5175 children | PM2.5, CO2, VOCs, O3, CO, radon, T/RH/comfort | Mixed; heterogeneous | Two-year cross-sectional campaign (harmonized protocol) |
| 18 | Subirana et al., 2024 | Spain (Catalonia) | Schools | 23 schools | CO2, NO2 | Predominantly natural / behavior-dependent | Three monitoring periods (Mar–Apr 2021; Oct 2021–Jan 2022; Mar–Jun 2022) |
| 28 | Villanueva et al., 2021 | Spain | Preschool, primary and secondary classrooms | 19 classrooms | CO2, PM2.5, PM10, UFP | Natural ventilation during reopening | 30 Sep–27 Oct 2020 |
| 29 | Rajagopalan et al., 2022 | Australia | School classrooms | 10 classrooms | CO2, PM2.5, T/RH, ventilation rates | Mixed / mechanically controlled classrooms | Year-long monitoring |
| 30 | Sivanantham et al., 2021 | France | Nursery and elementary schools | 602 classrooms; 308 schools | Aldehydes, VOC/SVOC, PM2.5, NO2, metals, fungal contamination | 73% without mechanical ventilation | One school week per classroom between 2013 and 2017 |
| 31 | Kim et al., 2021 | South Korea | Daycare center | 1 daycare center | Long-term indoor contaminants incl. PM, VOCs, CO2, T/RH | NR in abstract | Long-term monitoring |
| 32 | Park et al., 2022 | South Korea | Urban daycare center | 1 daycare center | PM10, PM2.5 | Closed windows / open windows / mechanical fans compared | 1 year |
| 33 | Konstantinou et al., 2022 | Cyprus | Primary schools | 42 schools | PM2.5, PM10, CO2, T/RH (indoor/outdoor) | Natural ventilation | May–Jul 2021 |
| 34 | Han et al., 2022 | South Korea | Elementary school classrooms | 2 schools; 3 classrooms tested repeatedly | PM2.5, PM10 | Air cleaner intervention in classrooms | Field tests across 8 air-cleaner models (campaign-based) |
| 35 | Zauli-Sajani et al., 2022 | Italy | Primary school classroom | 1 classroom + outdoor comparison | PM1, PM2.5, PM10, CO2, NO2 | Window/door opening protocol + air purifier | Alert-day monitoring campaign (pre-COVID) |
| 36 | Hama et al., 2023 | United Kingdom | Primary school classrooms | 30 classrooms; 5 schools | PM, CO2, T/RH, ventilation | Natural, single-sided and dual-sided patterns | Field monitoring campaign (NR in abstract) |
| 37 | De Coster et al., 2023 | Belgium | Daycare centres | 12 daycare centres | 47 VOCs, NO2, SO2, O3 (indoor/outdoor) | NR in abstract | One-week passive sampling in Sep and Jan |
| 38 | Chen et al., 2024 | USA | K–8 classrooms | 4 classrooms; 1 school | CO2, PM, PAC operation, T/RH | Mechanical ventilation + portable air cleaners | 7 weeks in 2022 |
| 39 | Xia et al., 2024 | USA | Naturally ventilated schools | 2 schools | BC, PM10, PM2.5, CO2, AER, T/RH, sound | Natural ventilation + portable air purifier intervention | Continuous campaign (NR in abstract) |
| 40 | Al Sharif et al., 2024 | Saudi Arabia | Public preschools | 4 preschools; 400 children | HCHO, VOCs, fine PM, CO2, T/RH | NR in abstract | Nov–Dec 2018 |
| 41 | Gilbey et al., 2024 | Australia | Childcare facilities | 22 centres | TVOC, HCHO, NO2, CO2, CO, PM fractions, UFP, T/RH | NR in abstract | 24-h monitoring in cold season and repeated in warm season |
| 42 | Wang et al., 2025 | USA | Elementary classrooms in K–8 school | 4 classrooms; 1 school | PM, CO2, T/RH | Mechanical ventilation; teacher decision patterns examined | 7 weeks |
| 43 | Du et al., 2025 | Switzerland | Primary schools | 24 schools (11 mechanically ventilated) | CO2, PM, VOCs; selected radon/nanoparticles | Natural vs mechanical ventilation | Four week-long campaigns from autumn 2021 to winter 2023 |
| 44 | Pérez-Moneo et al., 2025 | Spain | Primary school classrooms | 4 schools | PM2.5, VOCs, CO2, UFP and environmental characteristics | NR in abstract | Five campaigns of two weeks each |
| 45 | Collison et al., 2025 | Ireland | Naturally ventilated classrooms | 9 schools within 17 monitored buildings; 51 environments total | PM2.5, CO2, T/RH, TVOC, formaldehyde, BTEX, pinene, limonene, NO2 | Natural ventilation | Comprehensive multi-zone campaign (NR in abstract) |
| 46 | Hou et al., 2025 | China | Elementary school classrooms | 47 classrooms; 10 schools | T/RH, PM2.5, CO2, ventilation rate; short-term TVOC, HCHO, UFP | Predominantly natural/infiltration-based | Long-term monitoring Jan 2019–Jun 2021 |
| 47 | Charres et al., 2025 | Portugal | School with multiple education levels | Large school; exact classrooms NR in abstract | PM, gaseous pollutants, thermal comfort, microorganisms, ventilation | NR in abstract | Two seasonal campaigns (winter and spring) |
| 48 | Alves et al., 2025 | Angola | Primary schools | 4 schools | PM10, CO, CO2, TVOC, BTEX, O3, NO2, carbonyls, T/RH | Predominantly natural ventilation | Multi-day campaigns; passive + real-time monitoring |
| 49 | Fuentes-Ferragud et al., 2025 | Spain | Primary schools | 4 schools | Chemical pollutants, bioaerosols, respiratory viruses, VOC/SVOC non-targeted analysis | NR in abstract | Different seasons |
| 50 | Simona et al., 2025 | USA | Elementary school classrooms | 17 schools; 99 HEPA vs 87 control classrooms | PM2.5 / infiltration | Existing HVAC + portable HEPA vs sham units | Jul 2022–Jun 2023 |
| 51 | Meiss et al., 2021 | Spain | Nursery and primary school | Case study school; 10 teachers surveyed | CO2, TVOC, PM, hygrothermal comfort | Natural ventilation protocols / cross-ventilation | Pre-COVID winter week + post-COVID protocol scenarios in 2020–2021 |
